# The Neural Mechanisms of Associative Memory Revisited: fMRI Evidence from Implicit Contingency Learning

**DOI:** 10.3389/fpsyt.2019.01002

**Published:** 2020-02-03

**Authors:** Marco P. Caviezel, Carolin F. Reichert, Dena Sadeghi Bahmani, Christoph Linnemann, Caroline Liechti, Oliver Bieri, Stefan Borgwardt, Thomas Leyhe, Tobias Melcher

**Affiliations:** ^1^Center of Old Age Psychiatry, Psychiatric University Hospital (UPK), University of Basel, Basel, Switzerland; ^2^Transfaculty Research Platform Molecular and Cognitive Neuroscience, University of Basel, Basel, Switzerland; ^3^Centre for Chronobiology, Psychiatric University Hospital (UPK), University of Basel, Basel, Switzerland; ^4^Center of Affective, Stress and Sleep Disorders (ZASS), Psychiatric Clinics (UPK), University of Basel, Basel, Switzerland; ^5^Kermanshah University of Medical Sciences (KUMS), Substance Abuse Prevention Research Center, Health Institute, and Sleep Disorders Research Center, Kermanshah, Iran; ^6^Translational Psychiatry Unit (TPU), Department of Psychiatry and Psychotherapy, University of Lübeck, Lübeck, Germany; ^7^Division of Radiological Physics, Department of Radiology, University Hospital of Basel, University of Basel, Basel, Switzerland; ^8^Psychiatric University Hospital (UPK), University of Basel, Basel, Switzerland; ^9^Geriatric Psychiatry, Department of Geriatric Medicine FELIX PLATTER, University of Basel, Basel, Switzerland

**Keywords:** fMRI, functional connectivity, implicit memory, association memory, contingency learning, Alzheimer’s disease, default mode network, cingulate cortex

## Abstract

The literature describes a basic neurofunctional antagonism between episodic memory encoding and retrieval with opposed patterns of neural activation and deactivation, particularly in posterior midline regions. This has been coined the encoding/retrieval (E/R) flip. The present fMRI study uses an innovative task paradigm to further elucidate neurofunctional relations of encoding and retrieval in associative memory. Thereby, memory encoding is implemented as implicit (non-deliberate) cognitive process, whereas the prior literature focused mainly on explicit encoding. Moreover, instead of defining brain activations related to successful (vs. unsuccessful) memory performance, the task paradigm provides proper no-memory baseline conditions. More specifically, the encoding task includes trials with non-contingent (not learnable) stimulus combinations, while the retrieval task uses trials with a simple matching exercise with no mnemonic requirements. The analyses revealed circumscribed activation in the posterior middle cingulate cortex (pMCC) together with prominent deactivation in the anterior insula cortex (aIC) as core neural substrate of implicit memory encoding. Thereby, the pMCC exhibited positive functional connectivity to the hippocampus. Memory retrieval was related to an activation pattern exactly opposed to memory encoding with deactivation in the pMCC and activation in the aIC, while the aIC additionally exhibited a negative (i.e., arguably inhibitive) functional connectivity to the pMCC. Important to note, the observed pattern of activations/de-activations in the pMCC appears to conflict with prevalent E/R flip findings. The outlined results and their (alleged) discrepancies with prior study reports are discussed primarily in the context of the default mode network’s functioning and its context-sensitive regulation. Finally, we point out the relevance of the present work for the understanding and further investigation of the neurofunctional aberrations occurring during normal and pathological aging.

## Introduction

The aim of the present fMRI study was to further elucidate the neural mechanisms of the association memory (AM), separated into its constitutive complementary sub-processes of memory formation (i.e., encoding) and recall (i.e., retrieval). For this purpose, we adopted an innovative event-related fMRI task paradigm in which subjects are guided to acquire and later on retrieve arbitrary but contingent face–name combinations. Basically, AM can be conceived to be one of the most essential memory functions or even the most essential one. Thereby, it can be considered as the foundation for higher and more complex memory, and also other mental processes (1). By definition, AM is constituted by two interrelated aspects: first, the formation, and second, the recall of a cross-linking between separate (i.e., formerly unrelated) mental representations (1–4) becoming most obvious in the daily experience of memorizing (or possibly neglecting) a person’s name. Concerning the functional neuroanatomy, both AM encoding and retrieval are traditionally construed as ultimately relying on the medial temporal lobe (MTL), particularly the hippocampus and adjacent cortical regions (5–8). Newer research, however, shifts the focus to the functional contribution of other neo-cortical areas, particularly the so-called posterior midline region (PMR). The PMR comprises cortices of the posterior medial wall, particularly the posterior cingulate cortex (PCC) and precuneus (9–14) which shares dense reciprocal connections with the MTL (15–17). Of prime importance, prior fMRI studies consistently show opposed patterns of neural activation and deactivation in relation to mnemonic encoding and retrieval. Opposite levels of fMRI activity during encoding and retrieval have been primarily found in the PMR, which reliably exhibited activation during successful memory recall and deactivation during the preceding memory encoding (9, 11–14). This prominent finding, originally reported by Daselaar et al. (9), has been coined as the encoding/retrieval (E/R) flip and has been replicated in many studies [for review, see (12)]. Despite the robustness of the E/R flip, the underlying neural mechanisms are still not sufficiently understood. In the present study, we sought to further elucidate the neurofunctional antagonisms between mnemonic encoding and retrieval by changing the task conditions as they are prevalent in the E/R flip literature in three central respects:

- Explicit vs. implicit learning conditions: In the encoding tasks presented in the E/R flip literature, subjects are either explicitly instructed to learn the presented material or are aware of the following retrieval task (9, 11, 12, 14) [for an exception, see (13)]. These conditions that imply explicit learning do not apply to our new task paradigm, which was constructed to tap the neural mechanisms of implicit learning processes (i.e., learning without intention or awareness to do so), which we expected to exhibit a distinct neural signature. The investigation of implicit rather than explicit memory processes can be considered advantageous at least in two methodological respects: First, implicit memory tasks allow to define age- or dementia-related alterations of the neural memory function independent of working memory (WM) deficits, while a decreased WM capacity is well-known in both demented and non-demented older subjects (18, 19). Furthermore, implicit memory tasks can be expected to provide favorable testing conditions, because the subjective pressure to perform is reduced, which during explicit memory tasks could significantly impede performance, especially in older subjects with low self-efficacy expectation (20–22).
- Encoding-success vs. contingency effects: In the E/R flip literature, learning-related brain activations are defined as so-called encoding success effect (ESE). This effect is based on a subject-wise post-hoc coding of the experimental analysis conditions: One statistically contrasts trials of the learning phase comprising items which can be later on successfully retrieved against otherwise equivalent trials including items leading later on to a retrieval failure (23–25). Important to note, such differences in neural activation may only represent quantitative (rather than qualitative) process differences, because it cannot be excluded that non-retrieved items have been encoded before as well, at least to some extent. Following this reasoning, the ESE could possibly miss important brain activations underlying the acquisition of new memory associations as it eliminates relevant brain activations by contrasting events of the qualitatively same category. Therefore, in the present study we wanted to adopt a genuine non-learning baseline condition based on a manipulation of stimulus contingency. This methodological approach was already employed in one of our previous studies in which learning-related brain activations were defined in another theoretical context (26).
- Retrieval-success vs. no-memory baseline: Studies of the E/R flip literature define retrieval-related brain activations, similar to the ESE, as retrieval success effect (RSE), in that they contrast hits (correctly memorized items) against misses (falsely memorized items). RSE contrasts may again eliminate important memory-related brain activations, because misses arguably include appropriate retrieval efforts as well. Therefore, in our new paradigm we applied a proper “no-memory” baseline condition, which consists of a simple matching task (mock) without requirements on the memory system.

Taken together, the present study was designed to investigate the neurofunctional relations of encoding and retrieval in associative memory. While the previous literature on this topic has a strong focus on explicit learning processes, here we introduce a new task paradigm on mnemonic encoding and retrieval operationalized in the context of implicit contingency learning. That means, we pursued an extension of the E/R flip into the field of implicit memory, which assumably has the potential to add to the literature in an important way. More specifically, we expected to highlight new, i.e., so far neglected, aspects of the important involvement of the PMR in associative memory. Moreover, we expected to feature other brain activations and related functional connectivity importantly involved in associative memory (i.e., the E/R flip), but possibly ignored so far in the previous literature.

## Materials and Methods

### Participants

Twenty-two healthy subjects (mean age: 23.55, SD: 3.21, 16 females, 20 right-handed, mean school years 13.82, SD: 1.82), all native German speakers, with no personal and/or first-degree relative history of psychiatric or neurological disorders, participated in the study. All participants had normal or corrected-to-normal visual acuity and sufficient hearing ability. Most subjects were students recruited from the Faculty of Psychology of the University of Basel, Basel, Switzerland. All subjects gave written informed consent and were recompensed for their time spent by either participation hours (university course credits) or a financial incentive. The complete experimental procedure was approved by and conducted in accordance with the local ethic committee (EKNZ—Ethikkommission Nordwest- und Zentralschweiz; http://www.swissethics.ch).

### Procedure

Participants were welcomed at the scanner room of the University Hospital Basel (Clinic of Radiology and Nuclear Medicine) and went through a questionnaire regarding Magnetic resonance (MR) safety and their demographics. After lying in the scanner, the anatomical images were followed by the functional scans, first the encoding and second the retrieval run. Task instructions were prompted at the projector screen immediately before the start of the respective task. Afterwards, the subjects went through the written instructions, the subjects were asked orally trough a microphone system if they had further questions, which were addressed, if any have emerged. When subjects started with the first task (the encoding), they were not yet informed that a second task (the recall) would follow. After the scanning step, all participants explicitly confirmed that during the encoding task they had no expectation that they were expected to learn the names or to be asked about the names later on in the experiment.

### Stimulation and Experimental Task

The experimental task paradigm (stimulation and behavioral data acquisition) was implemented in E-Prime (Version 2.0, Psychology Software Tools, Pittsburgh, PA, USA) using two-sided headphones and a push-button panel. Visual stimuli were presented on a projector screen located at the end of the MR scanner and observed *via* a mirror system mounted on the head coil. The task paradigm consisted of two consecutive sub-tasks: first, to investigate the association learning (i.e., the encoding task); second, to investigate association recall (i.e., the retrieval task). Both tasks used an equivalent, stimulation consisting of face pictures (PNG files, black-and-white format; 22° visual angle) presented simultaneously with an auditory stimulus which was a spoken gender-matched name (WAV files). All depicted faces had an emotionally neutral expression and were taken from the life span database of adult facial stimuli (27, 28). The authors provided us with the respective picture files on request and gave us permission to adapt and use them for the present study purpose. Names were spoken by a neutral, artificial male voice and represented the most common names in German-speaking Switzerland for the given age groups (data provided by the Federal Statistical Office; http://www.bfs.admin.ch).

**Figure 1:**
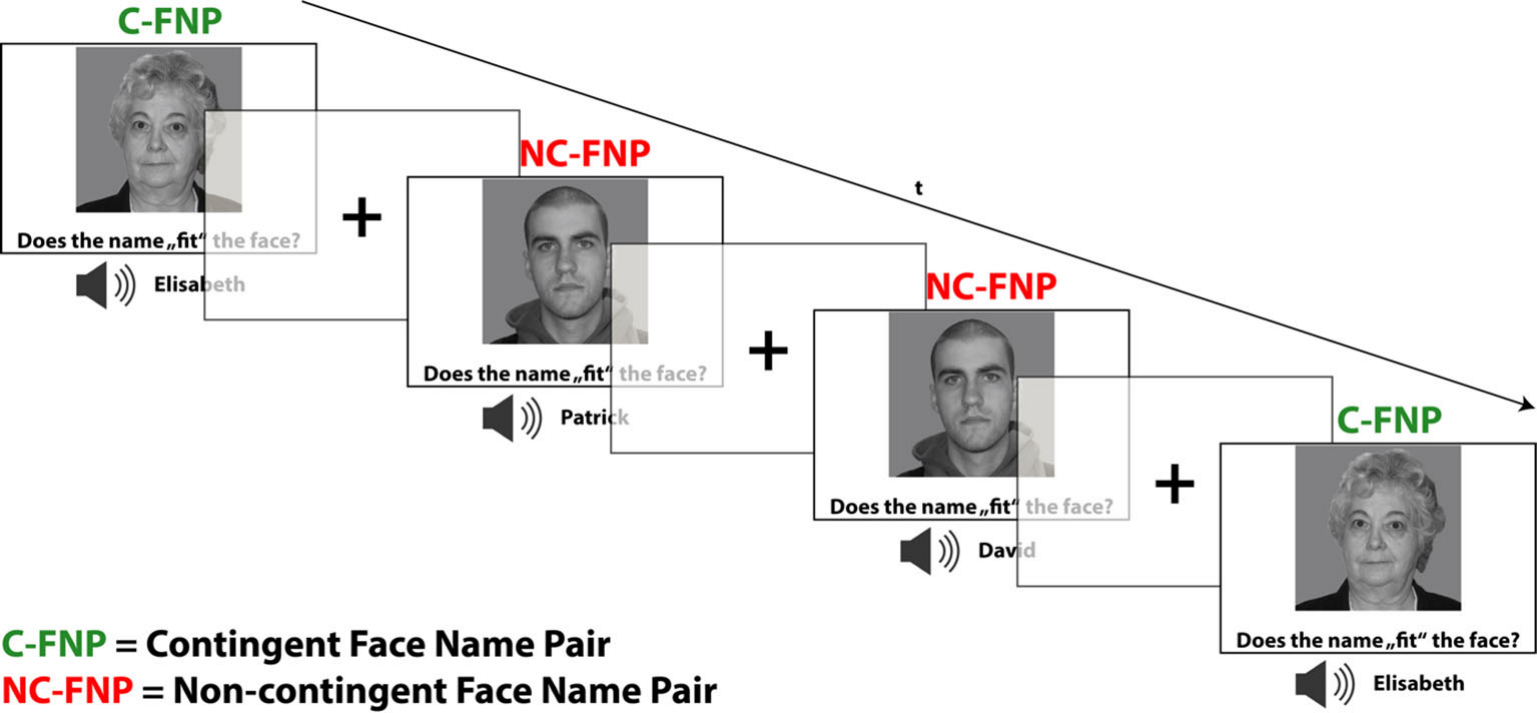
fMRI paradigm for memory encoding: implicit contingency learning task. Subjects rated the subjective fit of face–name pairs without instruction of learning. Half of the faces were presented in fixed, contingent combinations/face–name pairs (C-FNP), half were presented in varying, non-contingent combinations (NC-FNP). Duration of stimulus presentation was 2,800 ms. Trials were separated by a fixation cross with jittering ranging from 500 to 900 ms. The depicted faces were taken from the life span database of adult facial stimuli (27, 28).

The encoding task included a total of 12 different faces which were counterbalanced for sex and age (six young faces: mean age: 19.83 years, SD: 1.17; six old faces: mean age: 67.00 years, SD: 2.5). Given that the encoding task was planned to be kept implicit (according to the study purpose; see above), subjects were not instructed to remember the names occurring simultaneously with the faces. Instead, subjects were assigned a mock task in which they had to rate the subjective fit of the presented face–name combinations. Six out of 12 faces were presented in a fixed combination with a specific name. Those faces designed to provide a *contingency condition* which was expected to lead to association learning. Each face of the contingency condition was presented 12 times during the encoding task. The remaining six faces were likewise presented 12 times, and every time with a different name. Additionally, it was ensured that each name occurred in combination with each of the faces (avoiding sex mismatches). These trials thus provided a non-contingency, i.e., non-learning baseline condition, which appropriately controls for sensory, cognitive, and motor demands. A total of 72 contingent face–name pairs and 72 non-contingent face–name pairs were presented for 2,800 ms each, while button press responses were registered during the whole presentation time. Subjects were instructed to indicate a subjectively good fit by pressing with the right index finger (left response), whereas subjectively bad fits should be indicated by a middle finger press of the same hand (right response). The inter-stimulus interval comprised the presentation of a centered fixation cross and was systematically jittered from 500 to 900 ms to improve event separation and the efficiency of the hemodynamic response estimation (for trial composition and respective timing, see **Figure 1**). The trial sequence was counterbalanced for n−1 trial transitions, ensuring an equal number of condition and also response repetitions and switches.

In the subsequent retrieval task (of which the test subjects were unaware up to this point in time), only faces of the contingency condition were presented (three young faces: mean age: 19.33, SD: 1.5; three old faces: mean age: 66.00, SD 3.46). These familiar faces, however, occurred not only in combination with their established name, but also in combination with other names which were not part of the preceding encoding task. Therefore, the established contingent combinations were now resolved, and subjects had to confirm or falsify the combination at hand by an explicit memory recall. To provide an adequate “no-memory” baseline condition allowing to delineate retrieval-related brain activations, we presented participants with the same faces accompanied by either the word “man” or “woman” (spoken by the same voice). These words replaced the name words of the memory retrieval condition. Now, participants had to confirm or falsify whether the spoken word—in this case a sex labeling—corresponds to the depicted face. This matching task did not require a memory recall or other mnemonic process, therefore yielding an adequate baseline condition which adequately controls for non-mnemonic (motor, sensory, and other cognitive) processes.

**Figure 2:**
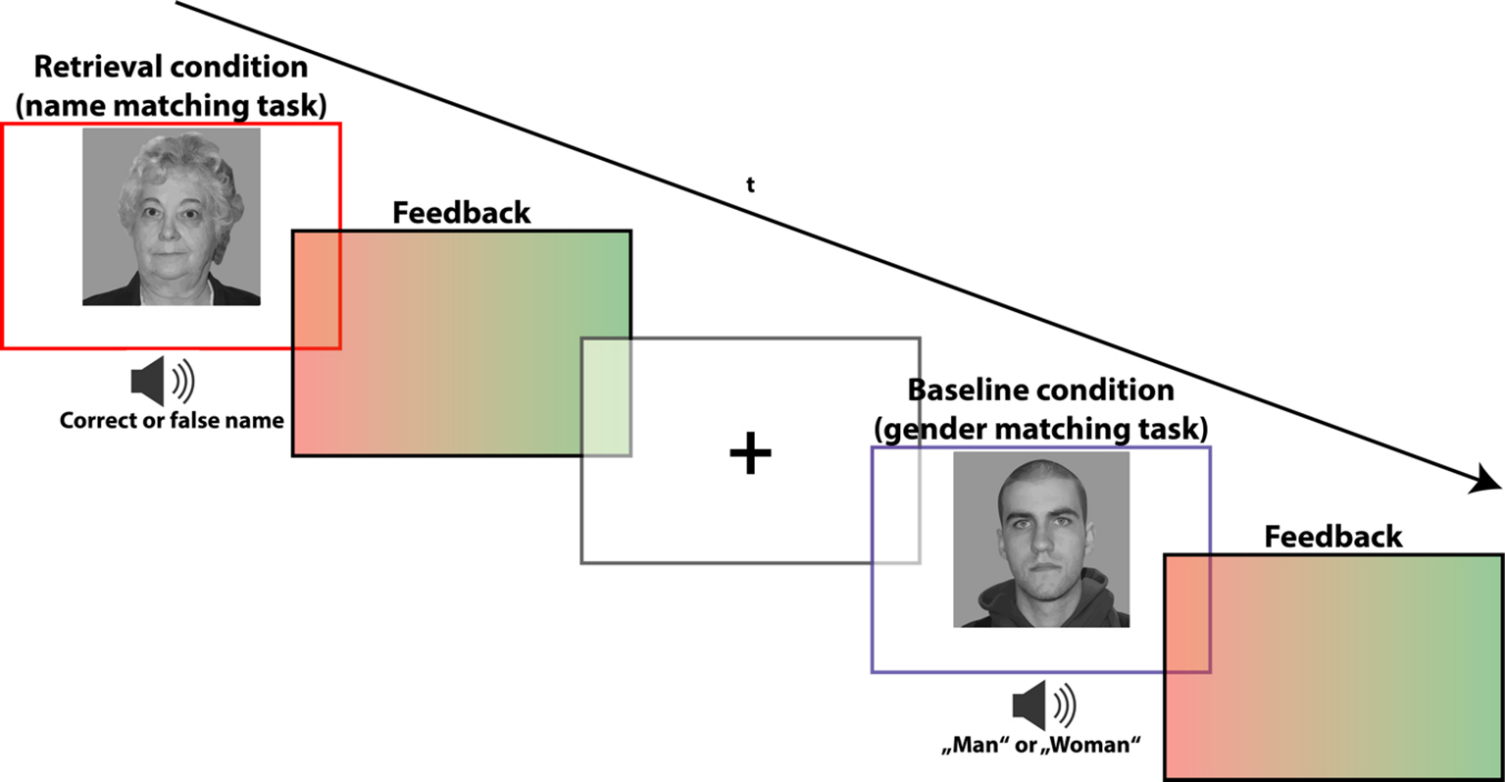
fMRI paradigm for memory retrieval: task to recall (i.e., verify) the names of the faces from the contingent combinations. During the genuine memory trials (indicated by a red frame), subjects were asked to indicate whether the spoken name indeed corresponds to the presented face, or not. During “no-memory” baseline trials (indicated by a blue frame), subjects were instructed to indicate whether the spoken sex label (“man” or “woman”) matches the face’s gender. Stimuli appeared for 2,500 ms and were followed by a visual feedback indicating a correct (green slide) or false (red slide) response. Trials were separated by a fixation cross with jittering ranging from 500 to 900 ms. The depicted faces were taken from the life span database of adult facial stimuli (22, 23).

The outlined experimental manipulation during the retrieval task lead to a total of four analysis conditions: a) memory retrieval—match (n = 36); b) memory retrieval—mismatch (n = 36); c) baseline—match (n = 18); d) baseline—mismatch (n = 18). After each matching decision, the participants received visual feedback for 300 ms, consisting of a green single-color patch indicating a correct response and red patch indicating a false response (for task trial constitution, see **Figure 2**). The trial sequence was counterbalanced for n-1 condition transition, while an equal number of matches and mismatches ensured an equal number of left and right button press responses, both within and across conditions. Trials with false responses were excluded from the further analyses. In addition to the neutral face stimuli, the retrieval task also included pictures of famous persons or celebrities, which did not appear during the preceding encoding task. The concerning task trials were excluded from the analyses because they are not encompassed by the scope of the present study.

### FMRI Data Acquisition and Image Preprocessing

A 3 Tesla MRI system was used (Magnetom Prisma, Siemens Healthcare, Erlangen, Germany), operating with a 20-channel head coil. Functional MRI acquisition was conducted with an interleaved T2*-weighted echo planar imaging sequence with 39 axial slices (3 mm), a field-of-view of 228 × 228 cm^2^, and an in-plane image matrix size of 76 × 76, giving a 3 × 3 × 3 mm^3^ spatial resolution. Images were acquired with the following parameters: 2,500 ms repetition time, 30 ms echo time, and 82° flip angle. The learning task required the acquisition of 216 scans, while the recall task included 323 brain volumes. All image preprocessing steps and further analyses were performed in SPM12 (http://www.fil.ion.ucl.ac.uk/spm/), running on MATLAB R2016b (The MathWorks, Natick, MA, USA). Field map distortion correction, slice time correction, realignment, co-registration, normalization to Montreal Neurological Institute (MNI) space, and smoothing (6 mm Gaussian kernel) was applied to the images using the default procedures and parameters of SPM12. None of the subjects exceeded our exclusion criteria of head movement greater than 3 mm or rotation greater than 3° (in either direction) during fMRI scanning. Based on the low to moderate motion parameters of the subjects, the preprocessing of the fMRI data did not go beyond the standard motion correction procedure (no exclusion of time points), while the realignment comprises the inclusion of the single-subject motion parameters as nuisance regressors in the first-level General linear model (GLM) (29, 30). For creation of figures of the neuroimaging results (brain renderings), we used the Python package Nilearn [(31), http://nilearn.github.io].

### fMRI Data Analysis

The experimental conditions were modeled on the basis of individual stimulus onset times using boxcar stimulus functions convolved with a canonical hemodynamic response function accounting for the delay of the BOLD (blood oxygen level–dependent) response. For both encoding and retrieval task, three movement parameters (mean: 2.83E−01 mm, SD: 6.82E−03) and three rotation parameters (mean: 4.82E−03°, SD: 6.82E−03) were considered as nuisance covariates on the individual subject level. Head motion of all participants was <2.0 mm, translation and rotation >2°

#### FMRI Contrast (Activation) Analyses

To define brain activations related to memory encoding, we contrasted trials of the contingency condition to trials of the non-contingency condition. The basic rationale behind this analysis is that no learning can occur when subjects are presented with non-contingent information. More specifically, in the non-contingency condition, learning should be precluded (or minimized) by the fact that every face is presented only once with a name and that each name occurs with every face. In other words, each possible face–name combination was presented exactly once so that evolving bindings should neutralize each other. The contrast between the contingency conditions has been calculated in both directions, with contrast “contingent minus non-contingent” and contrast “non-contingent minus contingent,” assumed to yield learning-related activations and deactivations, respectively.

Brain activations related to memory recall during the retrieval task have been defined by contrasting trials of the name-matching task versus trials of the sex-matching task. Again, the inverse contrast was computed to define retrieval-related deactivations.

For group statistics, whole brain random effect analyses were performed on the single subject contrast images by a non-parametrical testing method [10’000 permutations in SnPM13 toolbox, http://warwick.ac.uk/tenichols/snpm; (32)] as suggested by, e.g., Eklund et al. (33). Due to df < 20, variance smoothing was applied with an 8 mm FWHM Gaussian kernel. All reported results were thresholded at p < 0.05, Family-wise error (FWE)-corrected on peak level with a minimum cluster size of 15 contiguous voxels. Sex, age, handedness, and number of school years were included as covariates of no interest (i.e., nuisance variables) in all second-level analyses. To define a statistically significant overlap between the activation patterns of the different contrasts (representing memory encoding and retrieval), conjunction analyses were conducted following the minimum statistic (conjunction null) method, in which each of the included comparisons has to reach statistical significance (34).

#### Functional Connectivity Analyses

To define functional connectivity of the activated brain regions of the contrast analyses, we used the generalized psychophysiological interaction (gPPI) approach (35) in the gPPI toolbox of the SPM12. gPPIs have been shown to have an increased sensitivity and also specificity as compared to the standard PPI analysis procedure (35). The selection of seed regions for the gPPIs was determined *a priori* depending on the results of the conjunction analyses. Thereby, we planned to select those brain regions which exhibit an inverse activation pattern related to memory encoding and memory retrieval, i.e., constituting an E/R flip. At the statistical level, these regions should exhibit a significant overlap of positive activation in relation to the one and negative activation (i.e., deactivation) in relation to the other memory process. In this context, we were particularly interested in the functional connectivity of the PMR, which we strongly expected to exhibit antagonistic activation during encoding and retrieval. Seeds were defined as 6-mm-radius spheres around the peak voxel. The gPPI analyses thresholds were defined at p < 0.001 (uncorrected) with a minimum cluster size of 20 contiguous voxels. The fact that we used different statistical thresholds for the contrast analyses and the PPI analyses may appear as inconsistency, at least at first sight, but can be well explained. More specifically, PPIs use an interaction term as regressor to explain variance in brain signals. This interaction term consists of the convolved product of a physiological measurement series [deconvolved haemodynamic response function (HRF)] and a contrast vector. Contrast analyses basically only use a simple term (the convolved design vector) as regressor, so that they have an increased statistical power due to a decreased influence of error variance. Moreover, the regression model of PPI analyses includes three regressors (the psychological variable, the physiological variable, and their interaction), so that the variable of interest (the interaction term) has to compete with two further variables in the explanation of variance, which additionally decreases statistical power. Moreover, functional connectivity measures are particularly sensitive to artifacts due to participants’ head motion or modifications of physiological parameters decreasing the power of related statistical variables (36, 37). Therefore, one can argue that the reduced statistical power of PPIs actually requires some adaptation of the statistical threshold, in order to get comparable or commensurate results (38, 39). Nevertheless, the reduction of the statistical thresholding, together with the post-hoc definition of seed regions, gives the PPI analyses an exploratory character.

## Results

### Behavioral Data

In the encoding task, the proportion of fit to non-fit ratings did not differ significantly between contingent and non-contingent trials (contingent: M = 71.14, SD = 1.08; non-contingent: M = 71.40, SD = 1.79; difference: t = −0.689, p = 0.5, N = 22). In contrast, reaction times (RTs) to contingent trials compared to RTs to non-contingent trials were significantly faster (RT contingent: M = 1,100 ms, SD = 0.15; RT non-contingent: M = 1,330 ms, SD = .15; t = −8.40, p < 0.001, N = 22). During the recall task, trials of the retrieval condition compared to baseline exhibited both significantly increased RTs (RT retrieval: M = 1,130 ms, SD = 121.21; RT baseline: M = 970 ms, SD = 91.39; difference: t = 8.454, p = 0.001, N = 22) and a significantly reduced number of correct trials (correct responses retrieval: M = 32.7, SD = 2.65; correct responses baseline: M = 34.23, SD = 1.31; difference: t = −2.648, p = 0.015, N = 22). The correct response rate in both conditions was above 90%.

### Functional Imaging Results

In the first paragraph of this subsection, we present functional brain activations related to memory encoding, whereas the second paragraph reports brain activations related to memory retrieval. The third paragraph then reports findings of conjunction analyses carried out to define regions of significant overlap between encoding- and retrieval-related brain activations/deactivations. Brain regions showing concurrently positive activation related to one and deactivation related to the other process in the conjunction analyses (thus forming an “E/R flip”) served as seeds for gPPI analyses.

The corresponding functional connectivity findings are reported at the end of this subsection. In addition, we uploaded the NeuroImaging Data Model (NIDM) results (40) from both the contrast and gPPI analyses to NeuroVault.org (41) to make all results of our study publicly traceable (https://neurovault.org/collections/5067/).

#### Encoding-Related Brain Activation/Deactivation

**Figure 3:**
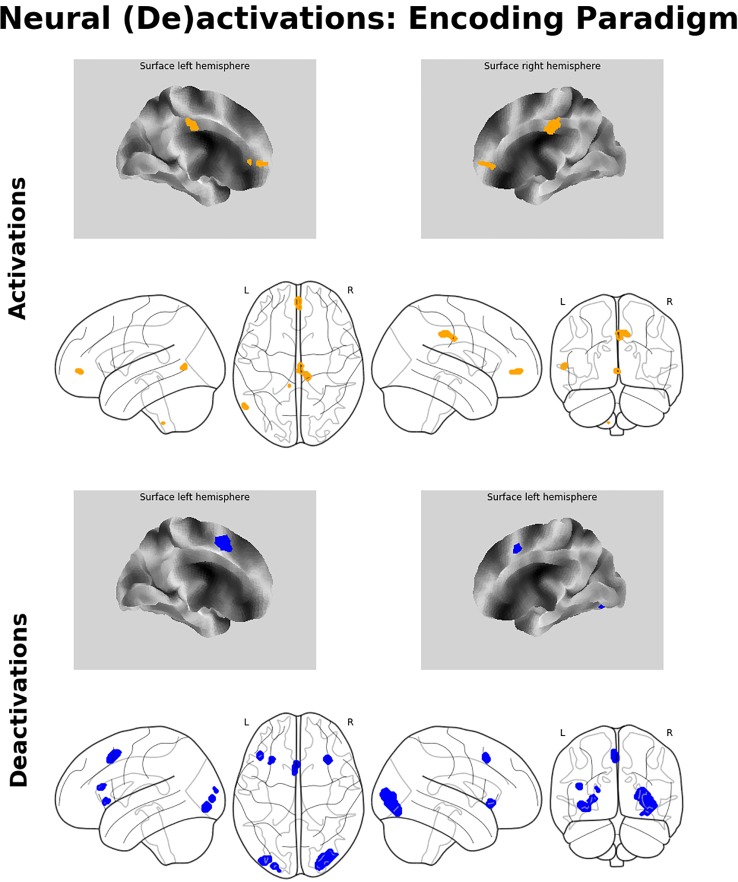
Neuroimaging findings. Medial wall surface renderings and glass brain images of brain activations (orange) and deactivations (blue) related to memory encoding. Depicted (de-)activations were thresholded at p_FWE_ < 0.05 at peak level with a minimum cluster size of 15 contiguous voxels.

The analysis of the fMRI data revealed two prominent “midline” activation clusters in relation to memory encoding, namely in the middle cingulate cortex (MCC) and in the frontal pole, partly reaching into the anterior cingulate cortex. Moreover, there was significant activation in the left middle temporal gyrus (MTG). Neural deactivations found in the inverse contrast occurred bilaterally in the anterior insular cortex (aIC), the inferior and middle occipital gyrus, in the posterior medial frontal gyrus, partly reaching into the supplementary motor area, as well as in the pars triangularis of the left inferior frontal gyrus (IFG). For a detailed listing and graphical depiction of the reported findings see **Table 1** and **Figure 3**.

**Table 1:** Significant neural activations and deactivations during mnemonic encoding and recall.

Task	Structure	Hem.	k	Pseudo t-Value	MNI Coordinates		
					x	y	z
**Learning**							
*Activations*	Frontal pole	m	61	6.3522	0	54	−2
	Middle temporal gyrus	l	37	6.3943	−59	−63	3
	Middle cingulate gyrus	r	116	6.2538	11	−29	39
*Deactivations*	Anterior insula lobe	r	93	7.1176	33	24	−2
		l	43	6.3739	−29	24	0
	IFG (p. triangularis)	l	54	6.4662	−41	29	15
	Posterior–medial frontal gyrus	l	164	6.3593	−3	12	53
	Inferior occipital gyrus	r	805	9.1249	35	−87	−3
	Middle occipital gyrus	l	178	6.8022	−39	−89	−6
		l	49	6.0699	−26	−93	3
**Recall**							
*Activations*	Anterior insula lobe	r	35	6.529	30	24	−5
*Deactivations*	Middle temporal gyrus	r	76	6.0881	60	−44	2
		l	1,000	7.1265	−44	−60	11
	Inferior temporal gyrus	r	79	6.2472	56	−57	−11
		r	33	6.17	50	−68	−9
	Middle cingulate gyrus	l	353	7.0322	−2	−17	41
		r	151	6.5413	12	−27	39
	Precuneus	l	57	7.125	−8	−54	69
	Middle occipital gyrus	r	52	6.1755	44	−74	14
		r	32	6.1062	39	−78	26

Since variance smoothing was applied due to df < 20, the values listed for learning and recall activation and deactivation don’t follow a t distribution. Using the Statistical nonParametrical Mapping (SnPM) toolbox (T. E. 32), the reported values are thresholded at P_FWE_ < 0.05 at peak level and with a minimum cluster size of 15 contiguous voxels. Hem., hemisphere; l, left; r, right; m, medial; coordinates are in Montreal Neurological Institute (MNI) space; k, cluster size; IFG, inferior frontal gyrus.

#### Retrieval-Related Brain Activation/Deactivation

**Figure 4:**
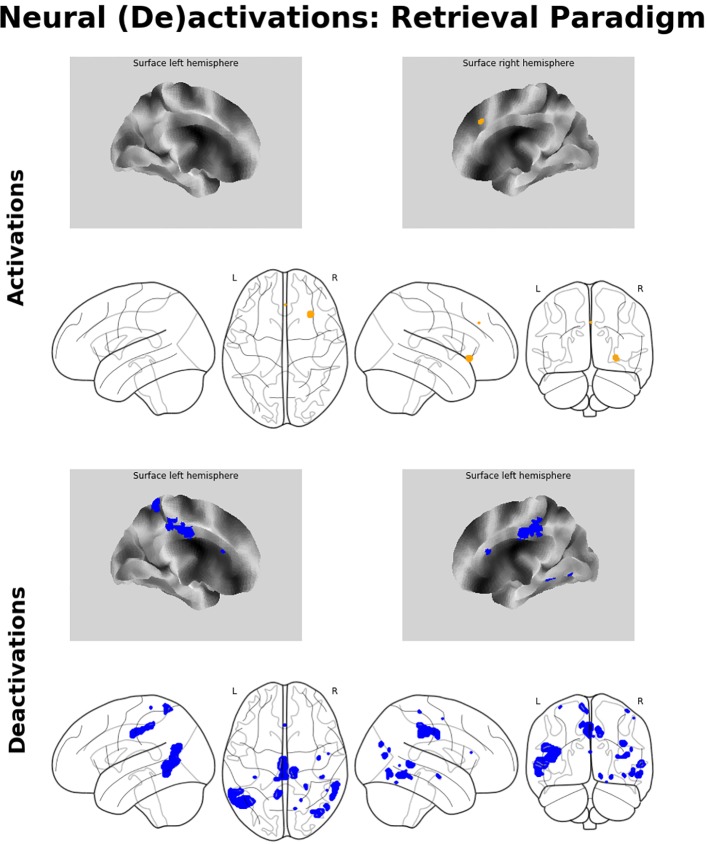
Neuroimaging findings. Medial wall surface renderings and glass brain images of brain activations (orange) and deactivations (blue) related to memory recall. Depicted (de-)activations were thresholded at p_FWE_ < 0.05 at peak level with a minimum cluster size of 15 contiguous voxels.

Memory retrieval was related to only one cluster of significant activation, which was located in the right aIC. In addition, several brain regions exhibited relative deactivation in the inverse contrast, which were the bilateral MCC and MTG, the right inferior temporal gyrus, partly reaching into the fusiform gyrus, the right middle occipital gyrus, and the left precuneus. For a detailed listing and graphical depiction of the retrieval-related brain activation/deactivation, see **Table 1** and **Figure 4**.

#### Overlap Between Encoding- and Retrieval-Related Activation/Deactivation

**Figure 5:**
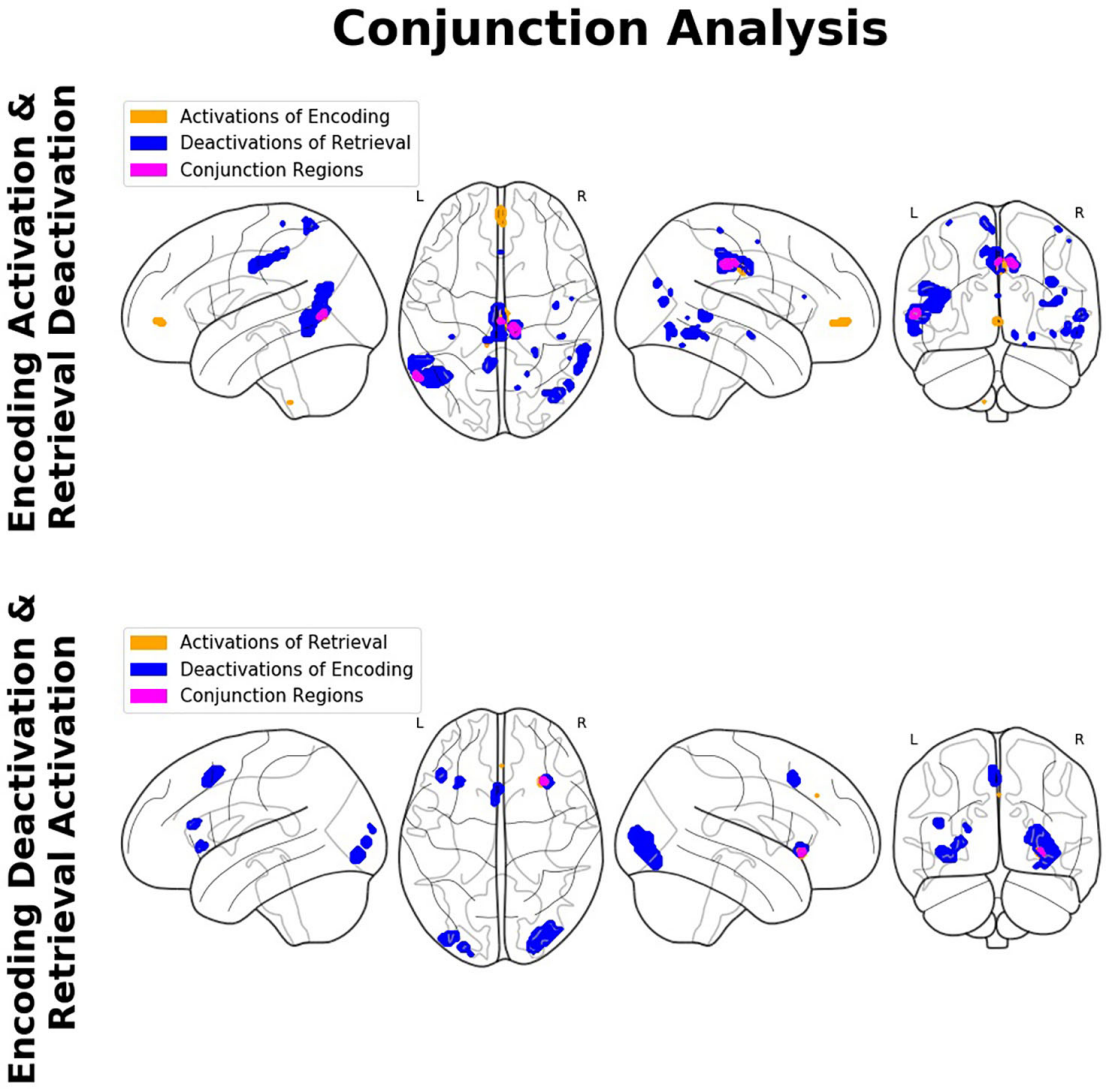
Findings of the conjunction analyses: Glass brain renderings of activations (orange) and deactivations (blue) and their overlap (magenta) in relation to memory encoding and retrieval. Regions showing an overlap of antagonistic activation related to the complementary mnemonic sub-processes (i.e., activation in relation to the one and deactivation in relation to the other) constitute a so-called encoding/flip (E/R) flip.

The conjunction analyses revealed significant overlap between retrieval-related activations and encoding-related deactivations (in terms of a genuine E/R flip as defined in the previous literature) in one single region, namely the right aIC. The overlap between encoding-related activations and retrieval-related deactivations (in terms of an “inverted” E/R flip) occurred in the right MCC and left MTG. The reported activation/deactivation clusters (coordinates and statistics) are listed in **Table 2** and graphically depicted in **Figure 5**.

**Table 2:** Overlapping regions of antagonistic activation (activation and deactivation) during mnemonic encoding and recall [i.e., regions forming an encoding/retrieval (E/R) flip].

Task	Structure	Hem.	k	Pseudo t-Value	MNI Coordinates		
					x	y	z
**E/R flip:**							
**overlapping**							
**brain regions**							
							
*Activation*							
*learning and*							
*deactivation*							
*recall*	Middle temporal gyrus	l	30		−57	−61	2
	Middle cingulate gyrus	r	48		14	−32	38
							
*Deactivation*							
*learning and*							
*activation*							
*recall*	Anterior insula lobe	r	10		33	23	−6
							

Statistically significant overlap between the activation patterns of the different contrasts (representing memory encoding and retrieval), conjunction analyses were conducted following the minimum statistic conjunction null method, in which each of the included comparisons has to be significant (T. 34). Reported values are thresholded at p_FWE_ < 0.05 at peak level and with a minimum cluster size of 15 contiguous voxels. Coordinates are in MNI space.

#### Functional Connectivity Analyses (gPPIs)

**Figure 6:**
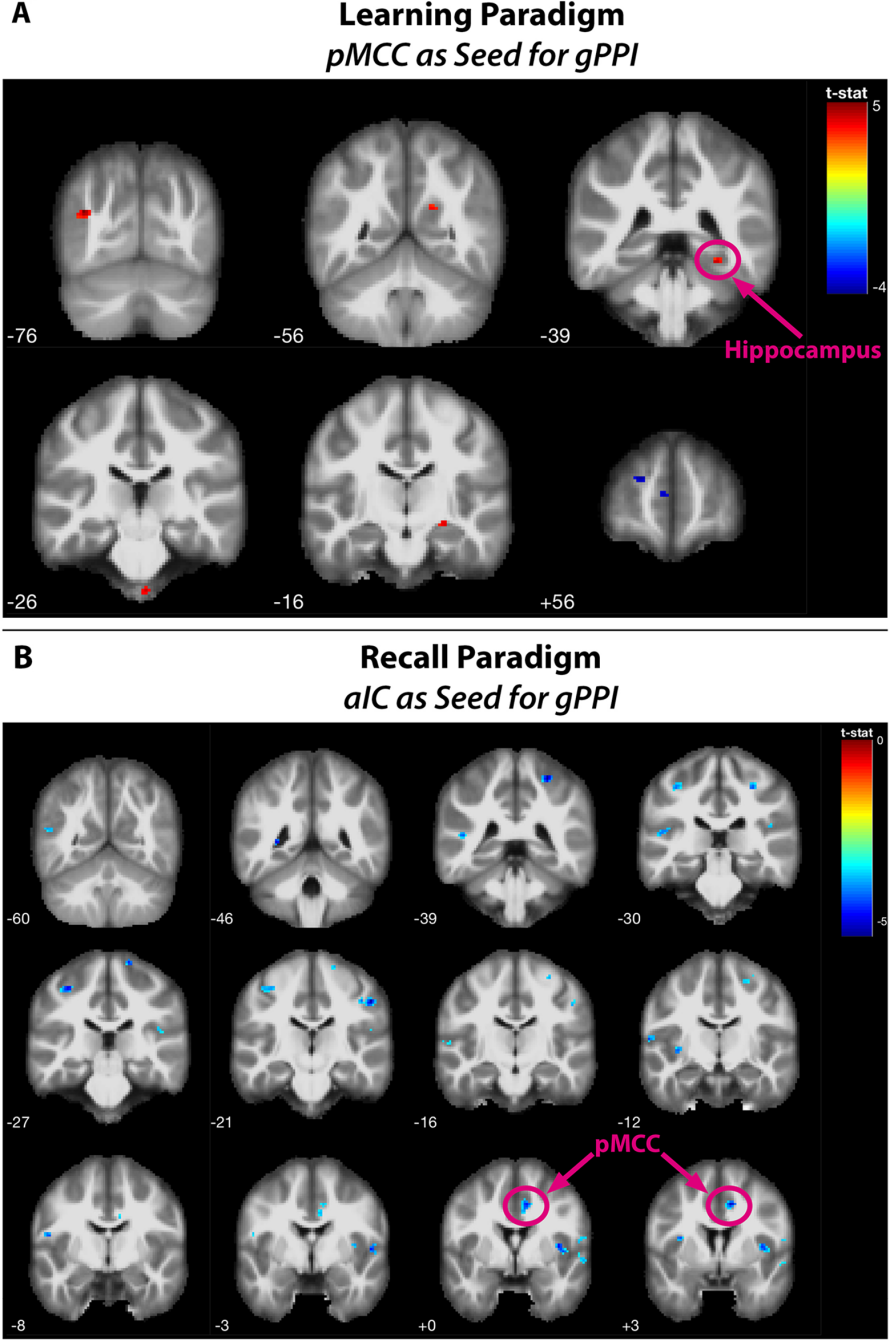
Findings of the generalized psychophysiological interaction (gPPI) analyses. Positive and negative functional connectivities during **(A)** the learning paradigm [seed is a 6-mm-radius sphere around the posterior midline region (PMR) as identified by the conjunction analysis (MNI: 14/−32/38)] and **(B)** the recall paradigm [seed is a 6-mm-radius sphere around the anterior insular cortex (aIC) as identified by the conjunction analysis (MNI: 33/23/−6)]. Functionally connected regions were rendered on coronal slices of the anatomic MNI template, thresholded at p < 0.001 with a minimum cluster size of 20 contiguous voxels.

In relation to memory encoding, the right MCC (MNI coordinates of the seed region: 14 −32 38) was positively connected with the right hippocampus, the right precuneus, the right fusiform gyrus, and the left middle occipital gyrus, whereas the left frontal pole exhibited a negative coupling. In the retrieval task, the right aIC (MNI coordinates: 33 23 −6) exhibited no significant positive connections at the given statistical threshold. Negative connections, on the other side, were revealed in a broader set of regions comprising of the bilateral aIC, the superior temporal gyrus and postcentral gyrus, the right MCC, the Rolandic operculum and precentral gyrus, as well as the left MTG and posterior insula lobe. For a detailed listing and graphical depiction of the reported functional connectivity findings see **Table 3** and **Figure 6**.

**Table 3:** Results of the functional connectivity analyses [generalized psychophysiological interaction (gPPI)].

Task	PPI Seed	Structure	Hem.	k	t-Value	MNI Coordinates		
						x	y	z
**Learning**	Right MCC (MNI: 14/−32/38)							
	*Positive connectivity*	Hippocampus	r	20	4.1883	26	−17	−12
		Fusiform gyrus	r	28	4.4662	30	−39	−14
		Precuneus	r	21	3.9252	18	−56	23
		Middle occipital gyrus	l	56	5.1585	−38	−77	20
		Brainstem	r	33	4.8104	3	−26	−57
	*Negative connectivity*	Frontal pole	l	47	−4.3544	−23	57	20
			l	24	−4.0078	−6	56	9
**Recall**	Right aIC (MNI: 33/23/−6)							
	*Positive connectivity*	n.s.						
	*Negative connectivity*	Anterior insula lobe	r	165	−5.1924	39	0	8
			l	23	−4.3677	−35	3	14
		Posterior insula lobe	l	25	−4.3259	−36	−12	0
		Superior temporal gyrus	r	135	−4.7395	53	−3	3
			l	61	−4.5079	−62	−12	11
			l	150	−4.8218	−48	−38	11
		Rolandic operculum	r	67	−4.1634	45	−29	18
		Middle temporal gyrus	l	46	−4.246	−51	−60	15
		Ventricle	l	28	−4.9826	−32	−47	5
		Middle cingulate gyrus	r	160	−5.4031	11	2	44
		Postcentral gyrus	r	155	−5.3258	51	−21	42
			r	106	−5.4224	29	−39	60
			r	27	−4.3616	30	−30	53
			l	225	−5.2522	−36	−26	56
			l	29	−4.3588	−53	−8	17
		Precentral gyrus	r	32	−4.6176	18	−27	78
			r	27	−3.8803	30	−17	65

Seed regions of the gPPIs were derived from the conjunction analyses’ findings. The reported values are thresholded at p < 0.001 uncorrected and with a minimum cluster size of 20 contiguous voxels. The lowered statistical threshold was applied because PPI analyses in general can be assumed to have a relatively high chance of false negatives compared to standard activation analyses (38, 39). MCC, middle cingulate cortex; aIC, anterior insular cortex. Coordinates are in MNI space. Hem., emisphere; l, left; r, right; coordinates are in MNI space; k, cluster size; n.s = not significant.

## Discussion

The present study was conducted to gain new insights into the mechanisms of the so-called E/R flip, which denotes the phenomenon that encoding and retrieval of the AM exhibit inverse patterns of neural activation and deactivation in central cortical regions (9, 11–14, 24, 42, 43). In this context, we were specifically interested in *implicit* memory encoding when subjects acquire new mnemonic associations effectively in an automatic manner, without explicit intention of learning. While previous studies on the E/R flip principally addressed *explicit* memory encoding, the analogous implicit encoding function very probably has—at least in part—a different functional neuroanatomy (44–46). Moreover, the neurofunctional dissociation between explicit and implicit AM encoding arguably spreads to the subsequent process of memory recall, in that the retrieval of implicitly learned associations and explicitly learned associations significantly deviate from each other, too (46, 47). To elucidate the expected neurofunctional specificities of encoding and retrieval of implicit memory associations, we adopted an innovative task paradigm which includes a dichotomous manipulation of stimulus contingency (contingent vs. non-contingent stimuli), thus allowing to define learning conditions (learning vs. non-learning baseline) during encoding independent of the later retrieval success. Thereby, we used stimulus compounds consisting of face–name pairs like well-established in the previous literature (7, 14).

### Behavioral Findings Confirm Mnemonic Processing

The purpose of the mock task during encoding (requiring a subjective matching decision concerning the face–name combinations) was merely to assure that participants attentively process the presented stimuli which include the to-be-learned associations. The faster response times during the contingency condition are most probably due to the repetition of identical face–name pairs, whereas the non-contingency condition (necessarily) exclusively includes unique face–name combinations. This response priming effect therefore affirms the occurrence of the intended learning process. During the recall task, the memory condition compared to the non-mnemonic baseline condition exhibited both an increased response time and a decreased rate of correct responses. Both behavioral effects are natural indicators of the cognitively more demanding and interference-prone memory process compared to the baseline condition (gender matching task).

### The PMR and its Functional Interactions During Memory Encoding and Retrieval

First and foremost, the neuroimaging findings clearly indicate a pivotal contribution of the PMR to the process of implicit association learning. The processing of the contingent information led to substantial activation in this region which, of note, appeared to be positively coupled with activation in hippocampal regions. Therefore, our data suggest that the PMR and its interaction with the hippocampal learning system represent an important part of the functional neuroanatomy of implicit association learning. This, however, does not apply to memory retrieval which in our data exhibited a different and partly opposed functional neuroanatomy. Important to note, the functional connectivity between the PMR and the MTL during encoding was restricted to the left hemisphere. The literature provides evidence that the lateralization of the hippocampal involvement in memory encoding is determined, among other things, by the verbalizability of the to-be-encoded material [e.g., (48, 49)]. According to this line of evidence, encoding of verbal material preferentially relies on left-hemispheric regions, whereas encoding of non-verbal (visual) information relies on right-hemispheric regions. Powell et al. (50) specifically report right-lateralized hippocampal activation for the encoding of face stimuli. Accordingly, we explain the connectivity to the right (rather than the left) hippocampus by the use of visual face stimuli in the encoding task. Facial stimuli, in our paradigm, represent the core of the to-be-encoded information, because the visual characteristics of faces are unique to the respective person, whereas names potentially also refer to other persons. Therefore, faces presumably represent the main reference in face–name memories.

**Figure A-1:**
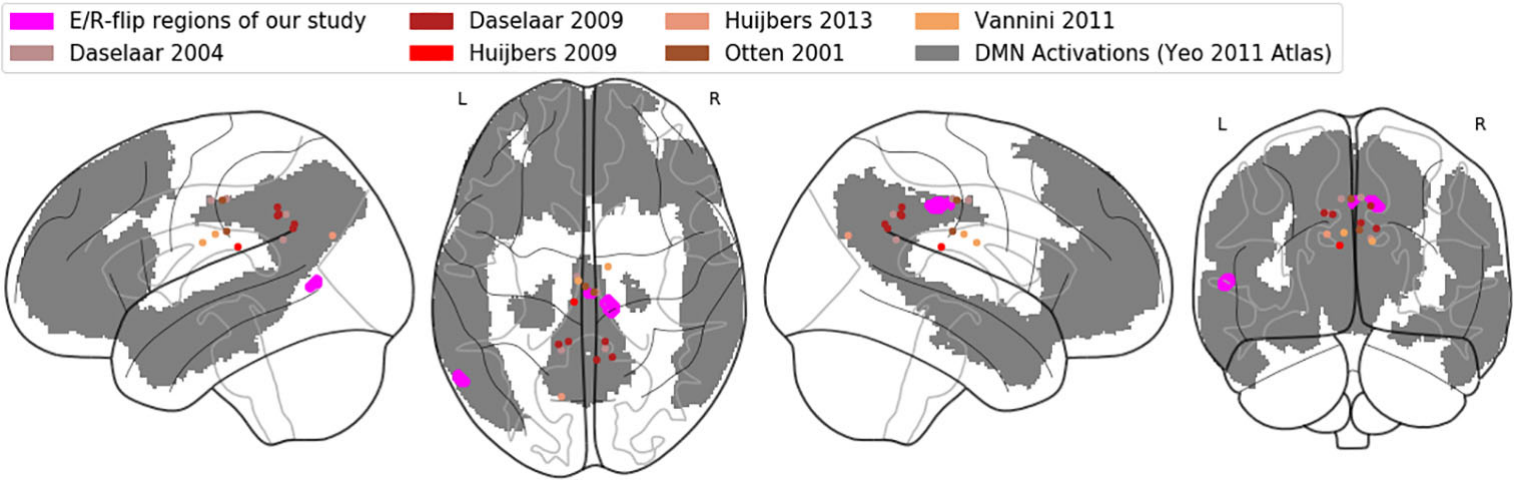
Comparative visualization of pMCC activation: Glass brain rendering of the pMCC activation of the conjunction analysis (magenta), together with (de-)activations labelled as PMR in previous studies (brown/red dots). The grey shaded areas represent the default-mode network in the atlas of Yeo (90).

Important to note at this point, the observed activation here labeled as PMR refers to the posterior midcingulate cortex (pMCC), while in the previous E/R flip literature, the PMR has been defined lying more posteriorly as part of the PCC, precuneus, and retro splenial cortex (9). In the Annex, we provide both a table of MNI coordinates (**Table A-1**) and a glass brain visualization (**Figure A-1**) of brain regions exhibiting an E/R flip and labeled as PMR in the studies of our reference list. Basically, the assorted coordinates exhibit a relative high variance along the anterior–posterior axis (ranging from y = −11 to y = −70), while about half of the listed activations lie even more anteriorly to our activation in the pMCC. Hence, our activation lies pretty in the center of what has been labeled the PMR in the previous literature. Moreover, while the peak of our activation lies in the MCC, the whole cluster considerably extends into the PCC and thereby overlaps with the more posteriorly located clusters of prior studies. The outlined spatial variance of posterior midline activations exhibiting an E/R flip indicates a need for future studies to focus on a further functional differentiation between sub-regions of the posterior midline cortex in the context of episodic memory processing. The literature includes already considerable evidence for functional subdivisions of the PMR, which arguably cannot be conceived as unitary neurofunctional unit (12).

**Table A-1:** Activations labelled as PMR in the prior literature. TAL converted to MNI using the Yale BioImage Suite Package (https://bioimagesuiteweb.github.io/webapp/mni2tal.html).

Study	Original coordinate space	MNI		
		X	Y	Z
10	TAL	-12	-49	36
		-5	-15	42
		8	-48	24
		4	-22	43
9	TAL	-13	-46	36
		11	-52	29
		4	-53	31
		8	-45	39
		-9	-45	35
13	MNI	-6	-27	21
11	MNI	-12	-70	26
19	TAL	-1	-20	42
		3	-22	28
14	TAL	-4	-17	27
		9	-11	23

TAL, Talairach; MNI, Montreal Neurological Institute.

Furthermore, we could relate the encoding process to a distinctive deactivation in the right aIC. Of prime importance, the PMR and the aIC exhibited inverse activation properties during the subsequent recall of the acquired associations (deactivation and activation, respectively); thus, form a double-sided E/R flip in our data. Well in line with the present findings, the aIC has been before related to the retrieval of both emotional and non-emotional memory contents, especially in recognition tasks (51–57). Moreover, the aIC has been consistently related to cue-induced drug craving and addictive behaviors in both human and animal studies (58, 59), which basically is a function of the associative memory, too. Together, the reported findings suggest an important integrative function of the aIC, which appears to be basically involved in the activation of associative connections between outer perceptions and inner representations. Thereby, the present study clearly corroborates that this function of the aIC is not restricted to the activation of emotional or interoceptive sensations, but likewise concerns less complex sensory or semantic representations like a person`s name, for instance. Of note, the present functional connectivity findings suggest that the opposed activation patterns in the aIC and the PMR during encoding and recall are driven—at least in part—by an inhibitory coupling between these regions. More specifically, we observed a negative functional connectivity between the aIC and the PMR during memory recall which presumably reflects an active downregulation or inhibition. In a broader context, this supposed neural inhibition may be part of the functional interaction between the salience network (SAL) and the default mode network (DMN) of which the aIC and the PMR represent primary hubs, respectively (60, 61). Basically, the function of SAL is to evaluate the personal relevance of a perceived stimulus or scene, which is achieved by recalling associated memories comprising both semantic information and affective or body states. This essential neurocognitive process provides the basis to detect salient events and to consequently adapt behaviorally to their related requirements (62, 63). Taken together, as primary hub of the SAL, the aIC can be conceived as dynamic interface unit mediating an adaptive switching between engagement and disengagement of different functional networks including the DMN. The observed negative connectivity between the aIC and PMR in the present work can be interpreted as one implementation of this general brain mechanism. The PMR is not only a primary hub of the DMN, but moreover has dense reciprocal connections with the medial temporal cortex, i.e., the hippocampal formation and adjacent parahippocampal cortices. Accordingly, the PMR can be considered as integrated part of the neural memory system, too (5, 6, 64, 65). However, so far, the mnemonic function of the PMR has been mainly described for memory retrieval processes and less for memory encoding or learning. More specifically, prior functional neuroimaging investigations relate regions corresponding to the PMR to the spontaneous (i.e., non-deliberate) activation of contextual associations, e.g., when subjects are presented with familiar (vs. unfamiliar) faces [e.g., (66–68)]. These studies strongly suggest a central role of the PMR in the retrieval of stored memory associations. In the prevalent literature on the E/R flip, the PMR is even construed to act as functional antagonist to AM encoding, which is based on its reliable deactivation related to this process as observed in a conclusive series of studies. The findings of the present study clearly challenge the generalizability of this notion, in that they very well support a functional involvement of the PMR—together with the hippocampus—in AM encoding. There are prior neuroimaging findings that likewise highlight PMR–hippocampal interactions as core neural substrate of successful memory formation (69). In the same sense, a series of human case studies and also experimental primate investigations report specific impairments in mnemonic encoding related to lesions in the PMR (70–74).

### Differences and (Alleged) Discrepancies With the Prior Literature

#### Dissociating Implicit and Explicit Processing Modes: Mnemonic Mechanisms Inside and Outside the Default Mode Network

The observed pattern of inverse activation in the PMR (and also the aIC) during memory encoding and memory retrieval essentially confirms the existence of a basic neurofunctional antagonism between these mnemonic sub-processes like established in the E/R flip literature. However, the pattern of PMR activation—positive activation during encoding and deactivation during retrieval—is exactly inverted with respect to the previous findings (12). The key difference between the previous studies and the present work to account for this (alleged) discrepancy are the different processing modes—implicit vs. explicit learning—which have been implemented during memory encoding. The implicit learning mode—subject of the present work—is characterized as keeping the learners unaware of learning, at least as long the encoding is not terminated. The explicit learning mode, which was used by most previous studies investigating the E/R flip (9, 11, 14, 43), is basically characterized by an involvement of WM and related to the central executive processing (45, 75, 76). We assume that these differential demands on WM capacities during implicit and explicit memory encoding basically determine the PMR functional involvement to this mnemonic process. Generally, WM activity is well known to decrease or even to interrupt activity in the DMN and particularly in the PMR (77–79). Accordingly, in a prior study, (80, 81) could already demonstrate sustained activity in regions of the DMN specifically during implicit mnemonic processing. In a similar sense, the implicit and the explicit processing modes can be related to different levels of cognitive effort, i.e., to a reduced cognitive effort for implicit learning. In this context, reduced levels of effort (or inversely increased levels of effort avoidance) have been related to increased activation in the DMN [e.g., (82)]. Accordingly, one may assume that the positive PMR activation during encoding in the present work is related—at least in part—to a decreased level of cognitive effort which subjects have to exert compared previous studies using explicit learning tasks.

Taken together, one may assume two parallel neural mechanisms for implicit and explicit memory formation which are basically executed inside and outside the DMN, involving PMR activation and deactivation. In contrast to the encoding task, AM retrieval in the present study was operationalized as explicit, deliberate and thus WM demanding process, which explains the observed deactivation in the PMR as hallmark of DMN downregulation. This finding is again well in line with the work of (80, 81) and also others (10, 83, 84) reporting substantial deactivation in the DMN, including the PMR and angular gyrus, during explicit cognitive processing and particularly explicit memory retrieval. In the following subsection, *Different Analytical Definitions of Retrieval-Related Brain Activations: Retrieval Success vs. Non-mnemonic Baseline*, we discuss that the retrieval-related activation in the PMR as consistently reported in prior studies may be an artifact of the applied analytical approach (“retrieval success effect”).

Prior neuroimaging studies on explicit memory retrieval consistently suggest a crucial role of ventrolateral prefrontal cortices together or connected with medial temporal regions [e.g., (85, 86)]. In the present study, however, we observed no prefrontal–hippocampal activations or connectivity in relation to memory retrieval. One plausible reason for this absence is that the adopted retrieval task bears relatively low control requirements, because the task does not require subjects to intrinsically recollect the names but rather “only” to recognize (i.e., to verify or falsify) them. Moreover, the high number of trial repetitions during the encoding has probably led to an overlearning, which is also supported by the low number of false responses in the behavioral data. Therefore, we assume that names in our study have been retrieved in a relatively automatic manner, which may underlie the absence of frontal–hippocampal involvement [cf. (87)].

In order to further corroborate our reasoning in which we relate our findings to the DMN’s functioning, we adopted the atlas of Yeo et al. (88) in order to confirm that the region in the pMCC exhibiting E/R flip-like activations/deactivations in the present work can be indeed considered as part of the DMN. **Figure A-1** in the Annex displays our activation in the pMCC (and also the PMR activations/deactivations of the referenced prior work) mapped on a glass brain, together with the DMN regions of the Yeo atlas. The graphic confirms that our activation in the pMCC indeed lies within the DMN.

#### Different Analytical Definitions of Encoding-Related Brain Activations: Encoding Success vs. Contingency Effects

The present study adopted a methodological innovation in the analysis of encoding-related brain activations. Basically, the prevalent E/R flip has been established by studies using the so-called *subsequent memory paradigm* (SMP). In the SMP, brain activations related to AM encoding are defined as *encoding success effect* (ESE), which uses a post-hoc coding of the analysis conditions based on the later retrieval performance in order to contrast retrieved vs. non-retrieved items. Of note, one could argue that this contrast computationally eliminates central encoding-related brain activations, because it cannot be excluded that non-retrieved items have involved the same encoding mechanism than retrieved items, at least to a lesser degree. Therefore, the literature on the E/R flip so far may neglect brain regions which are importantly engaged in the explicit acquisition of memory associations. In the present work, we replaced the ESE by a dichotomous manipulation of stimulus contingency (26), which allowed to create a genuine no-memory baseline condition. This approach allowed us to define pMCC activation and functional connectivity with the hippocampus as core neural substrate of implicit association learning. To our knowledge, it is desirable that future studies re-investigate the E/R flip for explicit AM encoding likewise using a contingent/non-contingent manipulation to provide an explicit non-learning baseline condition.

#### Different Analytical Definitions of Retrieval-Related Brain Activations: Retrieval Success vs. Non-Mnemonic Baseline

To define retrieval-related brain activations, prior studies commonly used the RSE, which—analogously to the ESE—contrasts hits (i.e., remembered items) versus misses (i.e., non-remembered items) (9, 11–14, 24, 43). The corresponding studies consistently report positive activation in the PMR as core neural substrate of AM retrieval, whereas the present work, on the contrary, found retrieval-related deactivation in this region. The core assumption of the applied contrast “remembered vs. not remembered” is that failed memory performance also implies failed, or at least reduced, functional activity in brain regions associated with the demanded memory process. This assumption may be challenged. More specifically, failed or impaired cognitive performance may be even related to increased activity in brain regions responsible for the demanded cognitive process, which can be considered as hallmark of the engagement of additional cognitive efforts during trials, which are especially difficult or error prone and which, hence, are related to performance impairments (89). Following this reasoning, retrieval failures (compared to hits) possibly lead to an even stronger activation in the neural memory system and thereby to an increased deactivation in the DMN during task engagement. Therefore, one may assume that the positive activation in the PMR related to explicit memory retrieval reported in prior studies may be related to the use of a baseline task condition that involves increased (rather than decreased) cognitive or memory effort and involves enhanced deactivation in the DMN. The use of a genuine “non-mnemonic” baseline condition like in the present work can help to prevent such limitations of the internal validity.

In the evaluation of the described findings and related conclusions, several limitations of the study should be noted and considered for future research. First, the present study may be considered limited principally by the relatively small sample size, so that replication in future studies using the same paradigm is warranted. At the same time, the study could apply a rather conservative statistical thresholding, which supports that the sample size was already sufficient for our purpose. Secondly, the study does not explicitly look for a potential modulation of results by the subjects’ gender. The reason for this neglect was that the number of male subjects in the sample was too small to get reliable results in this context. Third, given the superior number of female subjects, it would have been eligible to assess the menstrual cycle as a potential modulating variable. In this context, the literature includes evidence for a modulating influence of the menstrual cycle on both learning and memory processes as well as on related neural network activations [e.g., (90–92)].

## Conclusions and Outlook

**Figure 7:**
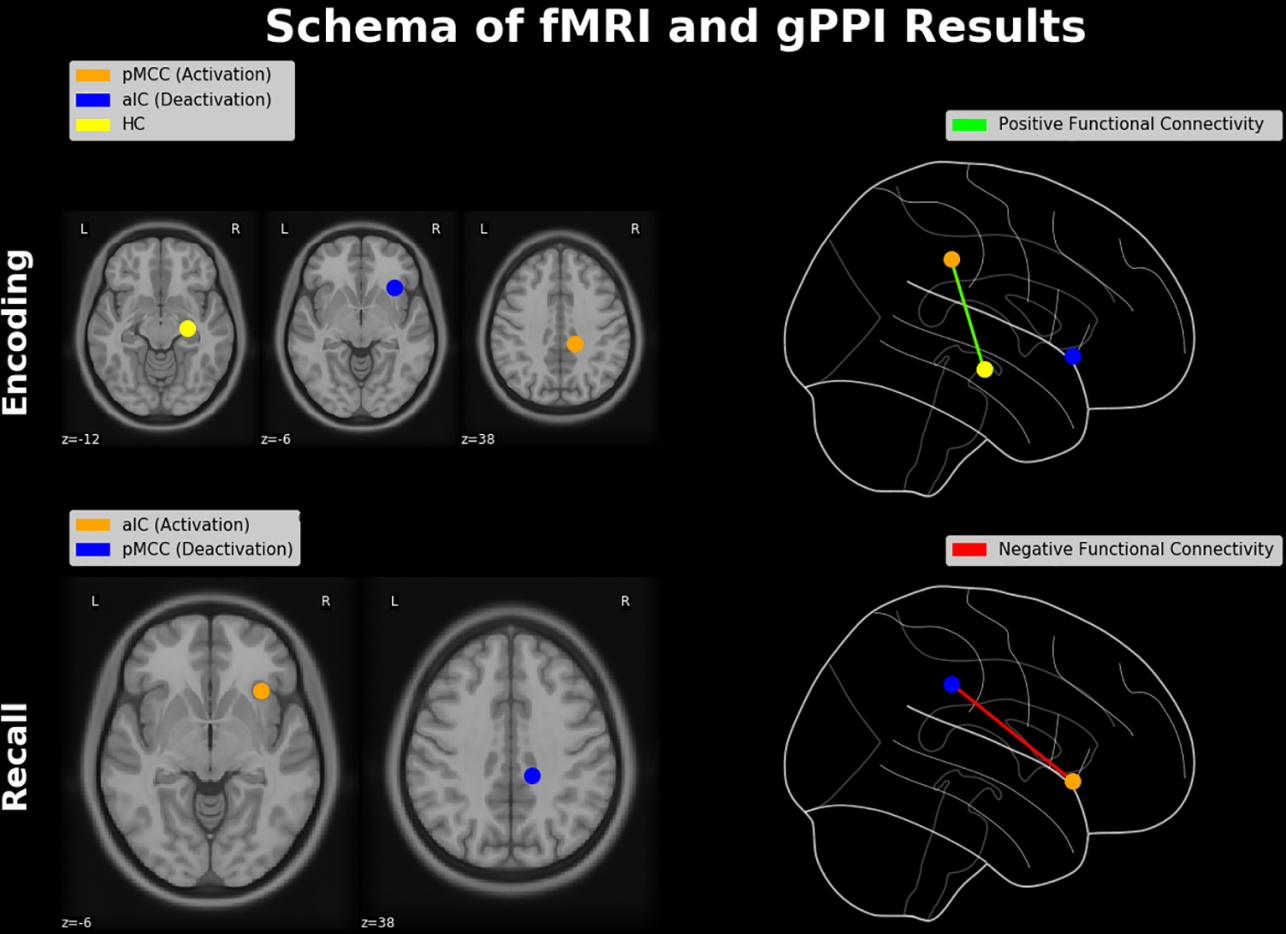
Schematic diagram of neuroimaging findings. Regions showing a pattern of antagonistic activation (i.e., activation and deactivation) during memory encoding and retrieval (constituting an E/R flip) and their functional connectivities. During encoding, activation in the pMCC is positively connected with the hippocampal formation, while the aIC is deactivated. During retrieval, the aIC is positively activated and thereby negatively connected with the PMR, which itself exhibits a significant deactivation. Brain regions were schematically rendered onto a T1 template (left-hand side) and projected in a glass brain (right-hand side). HC, hippocampus; pMCC, posterior middle cingulate cortex.

Taken together, in the present study we investigated the functional neuroanatomy of the AM with emphasis on the “neurofunctional antagonism” between mnemonic encoding and retrieval, coined as E/R flip in the previous literature. Contrary to prior E/R flip findings, encoding in our data exhibited substantial activation (rather than deactivation) in the PMR, which was positively connected with the hippocampus (see **Figure 7**, upper part). This alleged discrepancy of findings was traced back to the processing mode—implicit vs. explicit—of the implemented encoding process, which appears to generally moderate the functional involvement of the DMN in mnemonic processing. Of note, deactivation in the PMR during retrieval was negatively coupled with the aIC (see **Figure 7**, lower part), putatively reflecting an inhibitive regulating influence of the latter region on the DMN during explicit (i.e., deliberate) mnemonic or other higher order cognitive activity. The task paradigm introduced in the present work evidently provides access to the context-sensitive regulation of the DMN exerted by the aIC. Of note, the ability to downregulate the DMN and particularly activity in the PMR appears as significant marker in the early detection of AD well before manifest cognitive impairments occur (93–95). Thereby, this putative deficit in the adaptive regulation of the DMN in Alzheimer’s disease (AD) appears to be related, at least in part, to a disturbance of the aIC’s functional integrity (96–98). Based on the outlined findings, one may consider the paradigm of the present study as a promising tool for future studies to further elucidate the neurofunctional alterations or aberrations occurring both during healthy aging and in the course of neurodegenerative disease or dementia.

## Data Availability Statement

The raw data supporting the conclusions of this article will be made available by the authors, without undue reservation, to any qualified researcher. Unthresholded t-maps of this study can be found on NeuroVault.org (https://neurovault.org/collections/5067/).

## Ethics Statement

The studies involving human participants were reviewed and approved by EKNZ—Ethikkommission Nordwest- und Zentralschweiz. The patients/participants provided their written informed consent to participate in this study.

## Author Contributions

TM conceived and directed the project. MC and TM designed and planned the experiment. CR contributed to the development of the study design. MC, CaL, and CR conducted the data acquisition. MC conducted the preprocessing and analyzing of the data. TM supervised the preprocessing and analyzing of the data. MC and TM took the lead in writing the manuscript. All authors provided critical feedback and helped shape the research, analysis, and manuscript.

## Funding

This research was supported by grants of the German Research Foundation (DFG) to TM (ME 3082/6-1) and the Research Fund for Junior Researchers of the University of Basel (DMS2359) to CR.

## Conflict of Interest

The authors declare that the research was conducted in the absence of any commercial or financial relationships that could be construed as a potential conflict of interest.

## Appendix

We graphically compared the neuroanatomical position of the found activation in the pMCC (coordinates of the conjunction analysis) with activations labelled as PMR in the prior literature (referenced in the present study) by means of a common rendering on a glass brain. Reported coordinates were converted from Talairach to MNI space, if applicable. Basically, the displayed regions exhibit a relative high variance along the anterior-posterior axis ranging from y = -11 to y = -70, while about half of the reported coordinates lie anteriorly to our activation in the pMCC. Hence, our activation lies pretty in the center of what has been labeled as PMR in the prior literature. Moreover, we graphically tested as to whether the same activation in pMCC can be described as part of the default-mode network (DMN). For this purpose, we adopted the robust DMN atlas of Yeo (2011) and could confirm that the focus of our pMCC activation lies within of the network. Basically, the atlas of Yeo provides benchmark representations of brain regions and also neural networks and was derived from a mixture model of 1000 resting-state fMRI scans including connectivity and cluster analysis.
